# Lychee peel extract and chitosan synergistically delay mango ripening: Molecular insights

**DOI:** 10.1016/j.fochms.2026.100355

**Published:** 2026-01-13

**Authors:** Zhiwei Wu, Qinghua Qiao, Zhen Wang, Tiancui Shang, Shifang Wu, PengPeng He, Zhisheng Lin, Zhenxin Ren

**Affiliations:** a Xinjiang Key Laboratory of Lavender Conservation and Utilisation, College of Biological Sciences and Technology, Yili Normal University, Yining, 835000 Xinjiang, China; b Guangxi Key Laboratory for Agricultural Resources Chemistry and Biotechnology, College of Biology and Pharmacy, Yulin Normal University, Yulin 537000, Guangxi, China; c Renewable and Sustainable Energy, James Watt School of Engineering, University of Glasgow, Gilmorehill, Glasgow G128QQ, UK

**Keywords:** Postharvest preservation, Metabolomics, Transcriptomics, Synergistic mechanism, Fruit softening

## Abstract

Although lychee peel extract (LPE) is rich in bioactive compounds, its potential for postharvest fruit preservation remains unexplored. We hypothesised that LPE would act synergistically with chitosan (CH) to delay mango ripening by simultaneously modulating cell wall integrity, pigment metabolism, and hormone signaling pathways. Here, we demonstrate that chitosan combined with lychee peel extract (CHL) delays mango ripening through a multi-targeted mechanism. Specifically, CHL outperformed chitosan alone by significantly suppressing peel yellowing, maintaining fruit firmness, and reducing decay over 12 days of storage. Integrated transcriptomic and metabolomic analyses revealed that LPE reprogrammed ripening-associated pathways by (1) upregulating cell wall remodeling genes (CSLE1, XTH23) to stabilize pectin architecture, (2) retaining chlorophyll via suppressed CRTISO and PSY (carotenoid synthesis) and enhanced CHLP (chlorophyll biosynthesis), and (3) decoupling sugar-acid dynamics through γ-aminobutyric acid (GABA) and succinic acid accumulation. Notably, LPE attenuated ethylene-auxin- abscisic acid (ABA) crosstalk by downregulating ripening-specific transcription factors (ERF003, bZIPs) while activating stress-responsive WRKYs. These findings establish LPE as a sustainable alternative to synthetic preservatives, leveraging agricultural byproducts for eco-friendly fruit preservation.

## Highlights

• LPE-chitosan coating synergistically delayed mango ripening and reduced decay.
• Improved fruit firmness was linked to cell wall gene upregulation.
• Peel color retention was associated with modulated pigment metabolism pathways.
• Metabolic shifts toward organic acid accumulation helped maintain fruit acidity.
• Extended shelf life was correlated with altered plant hormone signaling crosstalk.

## 1 Introduction

Mango (*Mangifera indica* L.) is a globally cherished tropical fruit with high economic value, yet it suffers substantial postharvest losses (30–40%) due to rapid ripening and decay (Sivakumar et al., 2011). As a climacteric fruit, mango undergoes an irreversible surge in ethylene production postharvest, triggering a cascade of physiological changes including cell wall disassembly, chlorophyll degradation, carotenoid accumulation, and metabolic shifts, which collectively lead to softening, yellowing, and loss of edible quality (Bapat et al., 2010; Dautt-Castro et al., 2019). At the molecular level, ripening is orchestrated by a complex network where ethylene signaling activates transcription factors (e.g., ERFs) that upregulate cell wall-modifying enzymes (PG, PME, XTH), while chlorophyll degradation (SGR, NYC1) and carotenoid biosynthesis (PSY, CRTISO) govern peel color transition (Chen et al., 2022; Dang et al., 2023). Meanwhile, the dynamics of soluble solids and titratable acidity, influenced by sugar metabolism and organic acid accumulation, are critical indicators of ripening progression (Wu et al., 2024).

To mitigate these losses, various preservation strategies have been employed. Synthetic ethylene inhibitors such as 1-methylcyclopropene (1-MCP) are effective but raise concerns regarding chemical residues and application uniformity (Hofman et al., 2001). Chitosan (CH), a natural biopolymer, has gained popularity as an edible coating that primarily functions by forming a semi-permeable film and exhibiting antimicrobial properties (Ali et al., 2022; Mwakalesi & Umbayda, 2024). However, its single-target mechanism offers limited capacity to directly interfere with the host fruit's ripening-related gene expression and metabolic pathways (Wang, Wu, et al., 2024). Consequently, there is a growing pursuit of multi-target, natural alternatives that can simultaneously modulate physiology, metabolism, and defense responses.

While combining chitosan with bioactive plant extracts has become a prominent strategy to enhance preservation efficacy, the prevailing research perspective remains largely confined to evaluating the coating's external functionalities. These studies primarily focus on improved barrier properties, augmented antimicrobial activity (Ruan et al., 2022; Zhou et al., 2021), and resultant phenotypic outcomes such as reduced decay and weight loss (Gong et al., 2019; Sharma et al., 2022). Even when internal physiological shifts are examined, they are often limited to isolated enzyme activities (Shi et al., 2022) or single metabolic markers (Huang et al., 2023), lacking a systemic view. Consequently, a fundamental knowledge gap persists: whether such synergistic composites can transcend additive functional enhancements and actively orchestrate a systemic reprogramming of the host fruit's transcriptomic and metabolomic networks governing ripening.

Lychee peel extract (LPE) represents a uniquely promising candidate to address this gap. Unlike volatile essential oils that may impart undesirable odors (Freche et al., 2022; Zhou et al., 2021) or high-cost commercial polyphenols (Alav et al., 2024; Yang et al., 2022), LPE is a low-cost agricultural byproduct rich in stable oligomeric proanthocyanidins and flavonoids. Critically, proanthocyanidins have been implicated in modulating hormone signaling and cell wall dynamics in plant systems (Das et al., 2023; Xu et al., 2025). However, prior high-impact research on LPE has primarily explored its applications in green nanomaterial synthesis (Abdullah et al., 2024) or pharmacological models (Guo et al., 2025), leaving its potential for direct molecular-level intervention in fruit ripening virtually unexplored.

Therefore, we hypothesize that the integration of LPE with chitosan (CHL) will elicit a superior preservative effect by systemically reprogramming key ripening-associated pathways within mango fruit. To decode this internal synergy, we employed an integrated transcriptomic and metabolomic approach-a methodology notably absent in prior chitosan-extract studies (Wang, Yuan, et al., 2024). This study aims to: (i) physiologically validate the superiority of CHL over chitosan alone; (ii) identify LPE-driven metabolic shifts, particularly in organic acid homeostasis; and (iii) elucidate the transcriptional reprogramming of networks related to cell wall integrity, pigment metabolism, and hormone crosstalk.

## 2 Materials and methods

### 2.1 Preparation of chitosan and CHL solution

The chitosan solution was prepared as described by Poverenov et al. (2014) with modifications. Briefly, high molecular weight chitosan (molecular weight = 83 KD, viscosity less than 200 mPa-s, deacetylation degree 82.7%, Solarbio Co., Ltd., Beijing, China) was dissolved in distilled water containing 2% glacial acetic acid and heated to 55 °C to ensure complete dissolution, yielding a 1% (*w*/*v*) chitosan solution. To prepare the CHL solution, LPE (Virgin Biotech Co., Ltd., Xi'an, China) was added to the chitosan solution to create a composite coating solution. According to the detailed chemical profile provided by the manufacturer, the LPE powder used in this study is highly enriched in bioactive polyphenols (751 ± 22 mg GAE/g) and proanthocyanidins (285 ± 10 mg EE/g). The core bioactive monomers identified via liquid chromatography-tandem mass spectrometry (LC-MS/MS) include procyanidin A2 (23.5 ± 0.8 mg/g), procyanidin B2 (9.3 ± 0.2 mg/g), and epicatechin (3.6 ± 0.2 mg/g) (the complete chemical profile is presented in Supplementary Table S1).

### 2.2 Plant material and treatments

Mature green ‘*Tainong*’ mangoes (*Mangifera indica* L.) were harvested from a commercial orchard in Yulin, Guangxi Province, China. Uniform, defect-free fruits were selected, packed in plastic baskets with sanitary paper padding to prevent bruising, and transported to the laboratory at ambient temperature. Upon arrival, fruits were stored overnight at 25 °C (40–60% RH) to dissipate field heat.

The following day, fruits were washed, air-dried, and randomly allocated as intact units into three treatment groups: (1) control (CK, immersed in distilled water), (2) 1% chitosan (CH), and (3) 1% chitosan +0.5% LPE (CHL). Each group initially comprised 105 fruits. Groups were treated by immersion for 5 min in their respective solutions. After air-drying, fruits were stored at ambient temperature (80–95% RH, low light) for 12 days.

Experimental design and replication: The detailed sampling and replication strategy are summarized in Supplementary Fig. S1. At each sampling time point (0, 2, 4, 6, 8, 10, and 12 days), 15 fruits were randomly selected from each treatment group and divided into three biological replicates (*n* = 3). Each replicate consisted of five fruits.

For sensory evaluations (yellowing index and decay rate) and firmness: Each of the five fruits within a biological replicate was assessed individually. The results from the five fruits were then averaged to generate a single value representing that biological replicate.

For all other analyses (physicochemical, metabolomic, and transcriptomic): Pulp from the five fruits of each biological replicate were pooled to form one composite sample. This pooled sample was then flash-frozen in liquid nitrogen and stored at −80 °C. All measurements on these pooled samples were performed in triplicate as technical replicates.

The sampling time point for transcriptomic and metabolomic analyses (Day 4) was selected based on the physiological ripening characteristics of ‘*Tainong*’ mangoes. Previous studies on this cultivar have demonstrated that Day 4 represents a critical physiological transition period at 25 °C, characterized by the onset of rapid firmness loss and the initiation of the climacteric rise (Li, Li, Sun, Sheng and Tang, 2020, Li et al., 2023; Wu et al., 2025). By focusing on this window, we aimed to capture the early regulatory signals and molecular reprogramming events triggered by the CHL coating, which precede the macroscopic symptoms of senescence and decay (Zhou et al., 2025).

### 2.3 Determination of sensory quality

Sensory evaluations were performed on the individual fruits within each biological replicate as described in Section 2.2.

### 2.4 Fruit yellowing

The degree of yellowing in mango fruit was evaluated visually by categorizing samples into four groups: green, light yellow, yellow, and dark yellow. Fruits exhibiting light yellow, yellow, or dark yellow were classified as yellowed (Shao et al., 2017; Shi et al., 2022). The percentage of yellowed samples was calculated by dividing the number of yellowed fruits by the total number of samples, following the method described by Lihuan et al. (Shao et al., 2017).

### 2.5 Fruit decay and firmness evaluation

Fruit firmness was measured using a GY-4 digital fruit sclerometer (Tuopu Instrument Co., Ltd., Zhejiang, China). A 2 mm flat-head circular probe was inserted into the equatorial region of the fruit tissue at a constant speed over 10 mm, and the force required for penetration was recorded. This process was repeated three times at symmetrical points along the equator of each fruit, and each treatment was replicated three times with five fruits per replicate (Shi et al., 2022).

To assess fruit decay, visual evaluations were conducted on mangoes treated with CK (control, water), CH, and CHL at 0, 2, 4, 6, 8, 10, and 12 days. Fruits showing visible black rot lesions, skin collapse, or softening in the same region were classified as decayed (Shi et al., 2022). Five fruits were analyzed per treatment with three independent replicates. The decay rate was calculated using the formula:

$$\text{Decay}\;\left(\%\right)=\frac{\text{Number of decayed fruit}}{\text{Total number of fruit}}*100\%$$

### 2.6 Physicochemical properties measurement

Measurements were conducted on the pooled biological samples described in Section 2.2.

### 2.7 Chlorophyll and carotenoid content measurement

The contents of chlorophyll and carotenoids were determined using an ultraviolet spectrophotometer, following the method outlined by Chen et al. (Chen et al., 2022) with slight modifications. Approximately 0.5 g of fruit peel was homogenized with 80% acetone and extracted three times. The extract was then centrifuged at 12,000 ×*g* for 30 min. After centrifugation, the supernatant was combined with 80% acetone to a final volume of 10 mL. The concentrations of chlorophyll and carotenoids were calculated based on the absorbance readings at 470, 645, and 663 nm.

### 2.8 Measurement of soluble solids content

The soluble solids content (SSC) was determined following the method described by Liu et al. (Liu et al., 2017) with slight modifications. The fruit pulp was ground and filtered to obtain the filtrate. A handheld digital refractometer (Lichen, Shanghai, China) was used to measure the SSC. For each measurement, a pooled sample consisting of five fruits was used, and the experiments were conducted in triplicate to ensure reliable results.

### 2.9 Evaluation of titratable acidity

As previously detailed by Yin et al. (Yin et al., 2019), the sample solution underwent titration with a standardized 0.1 M sodium hydroxide solution. For each biological replicate, measurements were taken in triplicate (technical replicates) and averaged. Three independent biological replicates were analyzed per treatment (each biological replicate consisted of pooled samples from five fruits), and the data are presented as mean ± standard deviation. The calculation of titratable acid content was based on the following equation:

$$\text{Acid content}\;\left(\%\right)=\frac{V*N*K*B}{b*A}*100\%$$

V denotes the volume of sodium hydroxide solution consumed during the titration of the filtrate (in milliliters), N represents the concentration of the NaOH solution (in molarity, M), K is the conversion factor (0.064) derived using citric acid, B is the volume of the sample (in milliliters), b is the volume of filtrate utilized for titration (in milliliters), and A corresponds to the weight of the sample (in grams).

### 2.10 Extraction and quantification of pectin fractions

Pectin fractions were extracted from mango pulp (10 g) homogenized with 100 mL of 95% ethanol for 10 min. The residue was washed twice with 75% ethanol to obtain alcohol-insoluble solids (AIS). Water-soluble pectin (WSP) was extracted from 0.5 g AIS by incubation in water (25 °C, 2 h, stirring), followed by centrifugation (7000 rpm, 15 min, 4 °C). The supernatant was lyophilized. Chelator-soluble pectin (CSP) was extracted from the residue using 0.05 M CDTA (pH 5.2) with 0.05 M potassium acetate, repeated thrice. Hydrochloric acid-soluble pectin (HSP) was obtained by treating the residue with 0.5 M H₂SO₄ (85 °C, 1 h), followed by centrifugation. Pectin fractions were quantified via the Blumenkrantz method using D-galacturonic acid standards (Sigma-Aldrich, St. Louis, MO, USA). Data are presented as mean ± SD (*n* = 3 independent biological replicates).

### 2.11 Metabolome analysis

Analysis was performed on the pooled biological samples described in Section 2.2. Metabolomic analysis was conducted using an LC-ESI-MS/MS system (UPLC-QTRAP® 4500+) (Kangzhi Metabolomics Biotechnology Co., Wuhan, China) (Chen et al., 2013; Mo et al., 2019). Freeze-dried samples were homogenized to powder (mixer mill, 30 Hz, 1.5 min), and 0.1 g powder was extracted with 1.2 mL 70% methanol at 4 °C overnight (vortexed every 30 min). After centrifugation (12,000 ×*g*, 10 min), supernatants were filtered (0.22 μm) for LC-MS/MS.

Chromatographic separation employed a water-acetonitrile gradient (both with 0.1% formic acid) at 0.4 mL/min and 40 °C. Mass spectrometry operated in positive/negative ion modes (ESI Turbo Ion-Spray interface) with LIT and QQQ scans (Analyst 1.6.3 software). Differentially accumulated metabolites were defined by fold change ≥1.5 or ≤ 0.67 and VIP score ≥ 1 (PLS-DA model). Three biological replicates were analyzed per treatment.

### 2.12 RNA-seq

Analysis was performed on the pooled biological samples described in Section 2.2. Total RNA was extracted using a commercial kit and treated with DNase I to remove genomic DNA. RNA integrity and concentration were verified by 0.1% agarose gel electrophoresis and spectrophotometry (OD260/OD280). Sequencing libraries were prepared according to Patro et al. (Patro et al., 2014) and processed on the Illumina NovaSeq 6000 platform (Illumina, San Diego, CA, USA) with a paired-end 150 bp (PE150) sequencing strategy. Three biological replicates (five fruits per replicate) were performed for each treatment. The quality of raw data was assessed using FastQC, and clean reads were obtained by removing low-quality reads and adapters, ensuring Q30 > 92.0% and a stable GC content (44%–46%).

Clean reads were aligned to the mango reference genome (*Mangifera indica* ‘Hong Xiang Yao’ v1.0, NCBI BioProject: PRJNA487154) using HISAT2 (v2.1.0). Gene expression was quantified using StringTie (v1.3.3b) and normalized to Transcripts Per Million (TPM). Sample variability was assessed via principal component analysis (PCA) and PCoA. Differentially expressed genes (DEGs) were identified (DESeq2, v1.12.4; |log2FC| >2, *p* < 0.01) and functionally annotated using Gene Ontology (GO) and Kyoto Encyclopedia of Genes and Genomes (KEGG) pathways. Enriched terms (*p* < 0.05) were determined through hypergeometric testing.

### 2.13 Differential expression of hormone signal-related genes and their correlation with fruit ripening

To investigate whether the hormone-related DEGs induced by LPE treatment are associated with fruit ripening, we analyzed publicly available RNA-seq data from mango (*Tainong*) at key developmental and ripening stages (40, 60, 80 days after flowering (DAF), and 4, 8, 12 days after postharvest (DAP)) from the Sequence Read Archive (SRA, PRJNA697524, https://www.ncbi.nlm.nih.gov/sra/). FASTQ files were downloaded via SRA Explorer (https://sra-explorer.info) and quality-checked using FastQC. Reads were aligned to the mango reference genome using HISAT (Kim et al., 2015), and gene-level counts were calculated with HTSeq-count (Anders et al., 2015). DEGs were identified using DESeq2 (Love et al., 2014) with rigorous criteria (adjusted *p*-value ≤0.01, |log_2_FC| ≥ 1), ensuring reliable results across three biological replicates.

### 2.14 Quantitative real-time PCR (qRT-PCR) validation

qRT-PCR was performed following Ren et al. (2017). Total RNA was extracted using TRIzol reagent (Invitrogen, Carlsbad, USA) and treated with DNase I (Fermentas, Vilnius, Lithuania). First-strand cDNA was synthesized from 2 μg RNA using the RevertAid First Strand cDNA Synthesis Kit (Fermentas, Vilnius, Lithuania). Target gene expression was quantified using ACT as the endogenous control, and the primer sequences are provided in supplementary Table S2. Each sample was run in three technical replicates, and three biological replicates were used per treatment. Data are presented as means ± SE.

## 3 Results

Consistent with prior research findings (Abubakar Mshora et al., 2022), initial trials revealed that while 1% chitosan coating effectively reduced weight loss and maintained firmness in mangoes, higher concentrations triggered anaerobic respiration and quality deterioration. To optimize the treatment, LPE was incorporated into 1% chitosan coatings at concentrations of 0, 0.1, 0.25, 0.5, and 1.0%. Preliminary screening, assessing fruit firmness and flesh color, identified 0.5% and 1.0% LPE as most effective in delaying ripening. The 1% chitosan +0.5% LPE formulation demonstrated the most consistent and significant effects on ripening delay and quality retention, thus selected for further experiments (Data not shown). Subsequent experiments compared CH and CHL treatments against the control (CK), with CH as the baseline to isolate LPE-specific effects.

### 3.1 Phenotypic and physiological analysis

#### 3.1.1 CHL synergistically delays ripening and enhances postharvest quality

**Fig. 1:**
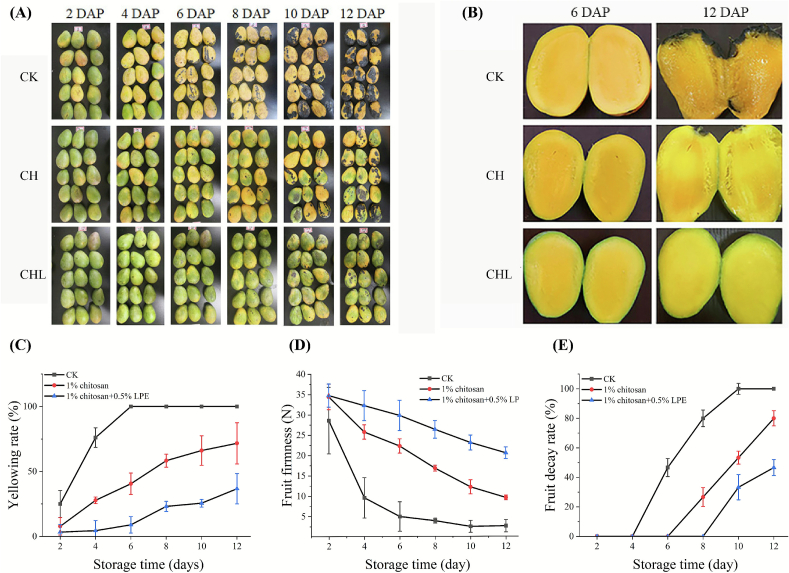
CHL treatment delays mango ripening during storage at 25 °C (A) Representative photographs of mango fruits from CK, CH, and CHL groups at 2-day intervals from 2 to 12 days after postharvest treatment (DAP). (B) Side-by-side comparison of fruit appearance at 6 and 12 DAP. (C) Percentage of fruits exhibiting yellowing (light yellow to dark yellow) during storage. (D) Fruit firmness, measured as penetration force (N), over the storage period. (E) Fruit decay incidence (%), assessed by visible rot or skin collapse. Data in (C)-(E) are presented as mean ± standard deviation (SD) of three independent biological replicates (each with five fruits).

During storage, CK (control, water-treated) mangoes exhibited rapid deterioration (Fig. 1A–B): the yellowing rates surged from 25% (day 2) to 100% (day 6) (Fig. 1C), firmness dropped drastically from 28.6 N to 2.7 N (Fig. 1D), and complete decay occurred by day 12 (Fig. 1E). CH treatment moderately slowed these trends, reducing yellowing to 71.7% and decay to 80% while maintaining a firmer texture (9.6 N). In contrast, CHL strongly suppressed yellowing to 36.7% (3.3-fold lower than CK), limited decay to 46.7%, and preserved firmness at 20.6 N (7.7-fold higher than CK). Notably, CHL delayed the onset of decay by 4 days (day 10 vs. day 6 in CK) and extended the retention of structural integrity 2.1-fold compared with CH alone (Fig. 1D–E). These results underscore the unique capacity of LPE to synergize with chitosan, providing superior decay suppression and texture preservation for prolonged shelf life.

#### 3.1.2 LPE decoupling sugar accumulation and acid depletion in fruit ripening

**Fig. 2:**
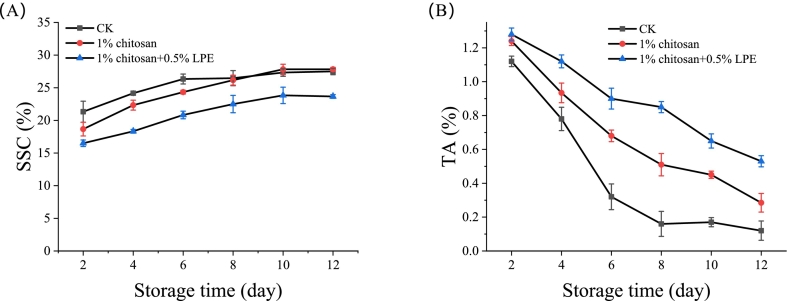
Disruption of sugar-acid dynamics and maintenance of metabolic homeostasis by LPE in mango fruit during storage (A) Soluble solids content (SSC, % Brix) and (B) titratable acidity (TA, % citric acid equivalent) in pulp of mangoes treated with CK, CH, or CHL over 12 days of storage at 25 °C. Data points represent the mean ± SD of three independent biological replicates (each replicate a pool of five fruits).

The contrasting dynamics of soluble solids content (SSC) and titratable acidity (TA) highlight LPE's unique role in stabilizing metabolic balance. While CK mangoes showed a sharp SSC rise (21.3% to 27.5%) and TA decline (1.12% to 0.12%) over 12 days (Fig. 2A-B), CH treatment only transiently slowed SSC accumulation (24.3% vs. CK's 26.3% at day 6), failing to sustain this effect. In stark contrast, CHL maintained long-term regulation, limiting SSC to 23.67% and preserving TA at 0.53% (4.4-fold higher than CK) by day 12. This striking divergence demonstrates LPE's ability to decouple sugar-acid balance dynamics, a feat unachieved by chitosan alone, effectively delaying ripening through sustained metabolic control.

#### 3.1.3 CHL outperforms CH in stabilizing sugar-acid balance and cell wall structure

**Fig. 3:**
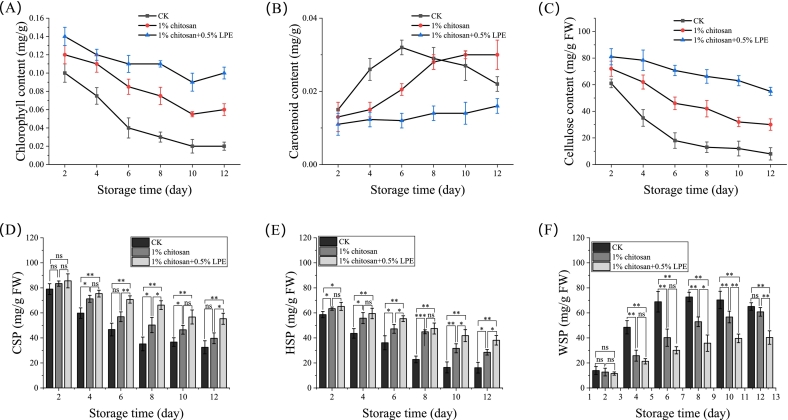
CHL stabilizes cell wall architecture and pigment metabolism in mango peel during storage (A) Chlorophyll content, (B) carotenoid content and (C) Cellulose content in fruit peel. (D—F) Content of different pectin fractions in fruit pulp: (D) WSP, (E) CSP, and (F) HSP. Data are presented as mean ± standard error (SE) of three independent biological replicates. Asterisks and “ns” above bars indicate the significance level of differences between specific treatment groups at the same time point (*p < 0.05, **p < 0.01, ns: not significant).

As shown in Fig. 3A–F, CHL surpassed CH in preserving cell wall integrity and delaying pigment changes. By day 12, CHL retained chlorophyll levels 4-fold higher than CK and 1.67-times higher than CH (Fig. 3A), while suppressing carotenoid buildup (Fig. 3B), effectively delaying peel yellowing. CHL also reduced WSP accumulation and slowed cellulose and CSP/HSP degradation (Fig. 3C–E). In contrast, CH showed only partial effects, failing to fully prevent pectin breakdown or carotenoid synthesis. These results demonstrate CHL's unique dual action: combining chitosan's baseline benefits with LPE-driven inhibition of chlorophyll loss, carotenoid accumulation, and cell wall disassembly-advantages unachievable by CH alone.

### 3.2 Metabolomic insights into LPE effects on mango fruit storage

To uncover how LPE enhances the preservative effect of chitosan, we compared the metabolomic profiles of CHL- and CH-treated fruit. Since the most pronounced physiological and biochemical differences were observed on day 4, while later stages were unsuitable for analysis due to decay and compromised quality, freeze-dried pulp samples from day 4 were selected for detailed investigation.

**Fig. 4:**
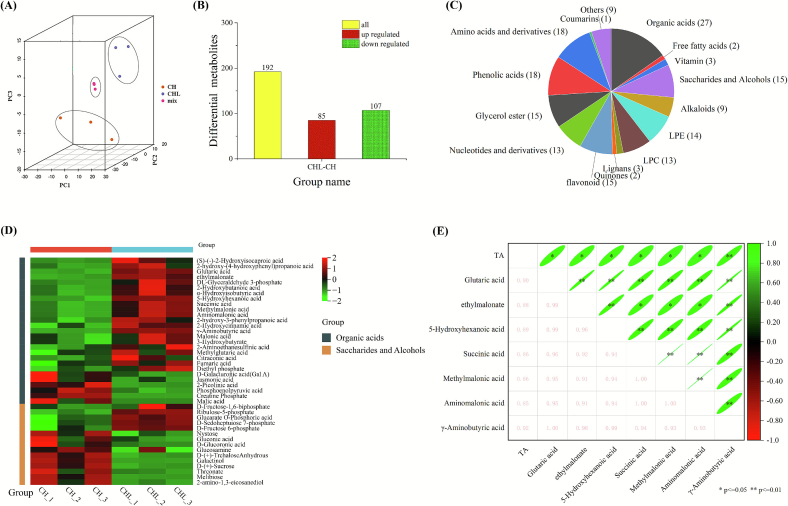
LPE reprograms organic acid metabolism to sustain fruit acidity (A) PCA score plot of metabolite profiles from CH- and CHL- treated mango pulp on day 4 of storage. (B) Bar chart showing the total number of differential metabolites (DMs) identified between CHL and CH, categorized as up- or down-regulated (fold change ≥1.5 or ≤ 0.67, VIP ≥ 1). (C) Pie chart displaying the classification and proportion of the identified DMs. (D) Hierarchical clustering heatmap of the relative abundance of differential organic acids, saccharides, and alcohols. The color scale represents *Z*-score normalized peak intensity (red, high; green, low). (E) Correlation matrix between the abundance of key organic acids and titratable acidity (TA). Ellipse color and shape represent the Pearson correlation coefficient (r), with asterisks indicating statistical significance (*p < 0.05, **p < 0.01, two-tailed test). All data are from three independent biological replicates and are presented as mean ± standard error (SE).

PCA revealed a clear separation between the metabolic profiles of the CH and CHL groups, with CHL forming a distinct cluster, indicating significant LPE-induced metabolic reprogramming (Fig. 4A). A total of 192 differential metabolites (DMs) were identified, with CHL exhibiting a distinct metabolic reprogramming compared to CH (Fig. 4B, C). Among these, organic acids were the most affected class (27 DMs), of which 21 showed marked upregulation, including glutaric acid, succinic acid, and GABA (Fig. 4D). Correlation analysis (Spearman, *p* < 0.05, |r| > 0.8) revealed that these organic acids, particularly GABA, were strongly correlated with titratable acidity (TA) (Fig. 4E). These findings suggest that LPE treatment promotes organic acid accumulation, thereby helping to maintain fruit acidity and quality during storage.

### 3.3 Transcriptomic analysis of CH and CHL treatments

**Fig. 5:**
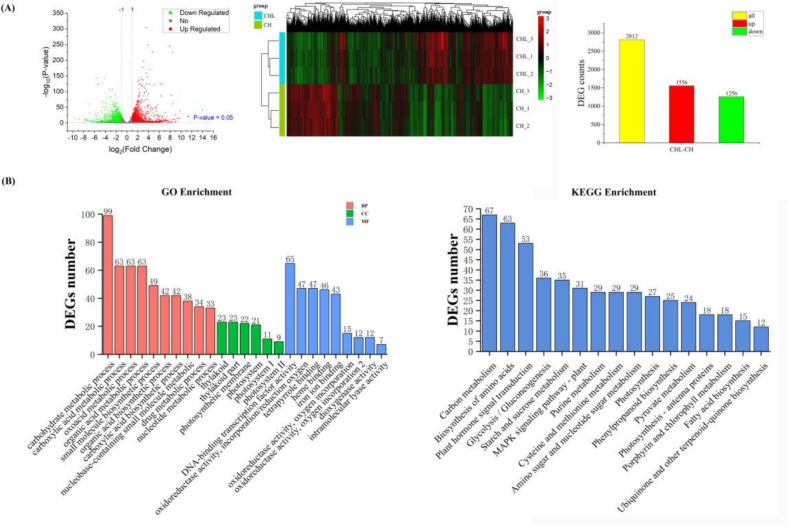
Transcriptional advantages of CHL over chitosan alone (A) Left: Volcano plot of differentially expressed genes (DEGs) between CHL and CH treatments on day 4 (|log2FC | > 2, p < 0.01). Middle: Hierarchical clustering heatmap of all DEGs, showing their expression patterns across three biological replicates per treatment. Right: Bar chart showing the number of up- and down-regulated DEGs. (B) GO enrichment analysis of DEGs, showing the top enriched terms in biological process, cellular component, and molecular function categories. (C) KEGG pathway enrichment analysis of DEGs, displaying the top 20 significantly enriched pathways. Analysis is based on three biological replicates per treatment.

Transcriptomic analysis revealed that CHL treatment induces significantly broader transcriptional changes than CH alone, which corresponded to its superior preservation efficacy. Compared to CH, CHL triggered differential expression of 2812 genes (1556 upregulated, 1256 downregulated) (Fig. 5A), highlighting LPE's unique ability to amplify chitosan's activity.

GO classification showed the most highly enriched DEGs belonged to biological processes such as carbohydrate metabolism (99 DEGs), carboxylic acid metabolism (63 DEGs), and organic acid metabolism (63 DEGs). For cellular components, DEGs were primarily associated with thylakoids (22 DEGs), thylakoid parts (22 DEGs), and photosynthetic membranes. Molecular function enrichment analysis revealed significant terms including DNA-binding transcription factor activity (65 DEGs), oxidoreductase activity (47 DEGs), tetrapyrrole binding (47 DEGs), and heme binding (46 DEGs) (Fig. 5B).

KEGG pathway analysis identified key metabolic pathways associated with ripening and storage quality, including glycolysis/gluconeogenesis (36 DEGs), starch and sucrose metabolism (35 DEGs), plant hormone signal transduction (53 DEGs)，and Porphyrin and chlorophyll metabolism (18 DEGs). These pathways are consistent with the physiological and metabolomic changes observed under LPE treatment, indicating that transcriptional reprogramming of metabolic and hormonal pathways contributes to enhanced fruit quality.

### 3.4 Co-expression analysis reveals DEGs associated with organic acid metabolism in LPE-treated mangoes

**Fig. 6:**
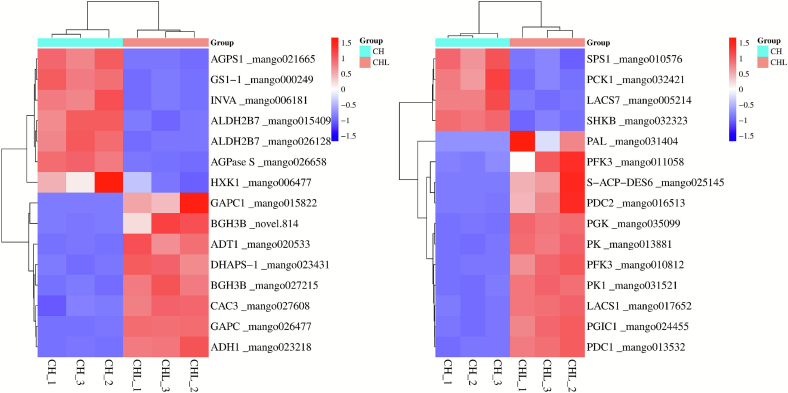
LPE enhances organic acid biosynthesis and suppresses sugar metabolism at the transcriptional level.

To explore the regulatory effects of LPE treatment on organic acid metabolism, 391 DEGs associated with 7 organic acid DMs were identified through Spearman correlation analysis (*p* < 0.05, |r| > 0.8). Subsequently, 30 DEGs were further filtered based on the criteria |log₂FC| > 2 and *p* ≤ 0.05 (Fig. 6). Among these, several key upregulated genes were identified, including *GAPC1* (mango015822), *PDC2* (mango016513), *PFK3* (mango010812, mango011058), *PK1* (mango031521), *DHAPS-1* (mango023431), *PGIC1* (mango024455), *CAC3* (mango027608), and *PDC1* (mango013532). These genes are primarily involved in organic acid biosynthesis, glycolysis, and the tricarboxylic acid (TCA) cycle, indicating that LPE treatment enhances energy metabolism and promotes organic acid accumulation. Conversely, downregulated genes included *PCK1* (mango032421), *HXK1* (mango006477), *SHKB* (mango032323), *SPS1* (mango010576), *AGPase S* (mango026658), and *AGPS1* (mango021665). These genes are associated with sugar metabolism and organic acid degradation, reflecting a coordinated metabolic shift favoring organic acid retention over their conversion from sugars. This regulatory pattern supports increased organic acid levels (including GABA, succinic acid, and glutaric acid), which contributes to enhanced fruit acidity and delayed ripening.

Expression patterns of 30 key DEGs strongly correlated (|R^2^| > 0.8, p < 0.05) with differential organic acid metabolites. Genes are involved in glycolysis, tricarboxylic acid (TCA) cycle, and sugar metabolism. Red squares indicate upregulation in CHL compared to CH, and blue squares indicate downregulation. Data are log2-transformed fold changes (CHL/CH) from three biological replicates. Key gene abbreviations: glyceraldehyde-3-phosphate dehydrogenase C1 (GAPC1), pyruvate decarboxylase 2 (PDC2), phosphofructokinase 3 (PFK3), pyruvate kinase 1 (PK1), phosphoglycerate kinase 1 (PGIC1), cytosolic aconitase 3 (CAC3), phosphoenolpyruvate carboxykinase 1 (PCK1), hexokinase 1 (HXK1), sucrose phosphate synthase 1 (SPS1), ADP-glucose pyrophosphorylase small subunit (AGPase S).

### 3.5 Differential regulation of peel color and fruit firmness through metabolic pathway- specific gene expression

**Fig. 7:**
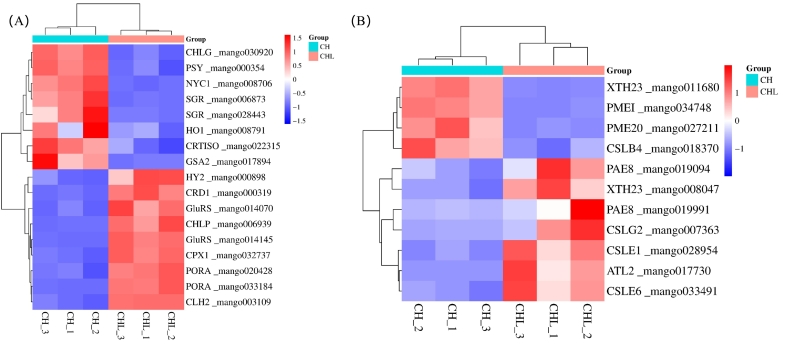
LPE stabilizes chlorophyll and cell walls via transcriptional regulation of specific metabolic pathways. (A) Heatmap showing expression of DEGs involved in porphyrin and chlorophyll metabolism, carotenoid biosynthesis, and chlorophyll degradation. Key genes: chlorophyll synthase (CHLG), protochlorophyllide oxidoreductase (PORA), chlorophyllase (CHLP), stay-green protein (SGR), carotenoid isomerase (CRTISO), phytoene synthase (PSY). (B) Heatmap showing expression of DEGs associated with cell wall metabolism, including genes for hemicellulose and pectin remodeling. Key genes: cellulose synthase-like E1 and E6 (CSLE1, CSLE6), xyloglucan endotransglucosylase/hydrolase 23 (XTH23), pectin methylesterase 20 and its inhibitor (PME20, PMEI). Data are z-score normalized log2(TPM + 1) values from three replicates. Red indicates upregulation in CHL vs. CH, blue indicates downregulation.

A comprehensive analysis of DEGs revealed key regulatory mechanisms underlying peel color and fruit firmness in response to treatment (Fig. 7A, B). A total of 15 DEGs associated with porphyrin and chlorophyll metabolism, 2 DEGs linked to carotenoid biosynthesis, and 11 markedly regulated cell wall metabolism-related DEGs (*p* ≤ 0.01, |log_2_FC| ≥ 2) were identified, highlighting a complex interplay of pathways modulating fruit quality.

In the porphyrin and chlorophyll metabolism pathway, several key genes promoting chlorophyll biosynthesis and maintenance, such as *CHLP* (mango006939), *PORA* (mango033184, mango020428), *CRD1* (mango000319), *CPX1* (mango032737), and *HY2* (mango000898), were upregulated. By contrast, *CHLG* (mango030920), which acts in later stages of chlorophyll synthesis, was downregulated, implying a shift in metabolic priority toward chlorophyll stability rather than active synthesis. Furthermore, the downregulation of chlorophyll degradation genes such as *SGR* (mango006873, mango028443) and *NYC1* (mango008706) likely delayed the breakdown of chlorophyll, thereby helping retain green coloration and delay peel yellowing. In the carotenoid biosynthesis pathway, *CRTISO* (mango022315) and *PSY* (mango000354) were downregulated. This suppression of carotenoid synthesis aligns with the delayed color transition from green to yellow, further supporting the treatment-mediated delay in ripening.

Regarding cell wall metabolism, the identified DEGs reflect a dynamic, finely tuned regulatory program that promotes firmness. Upregulated genes including *ATL2* (mango017730), *CSLG2* (mango007363), *CSLE1* (mango028954), *CSLE6* (mango033491), *PAE8* (mango019991, mango019094), and *XTH23* (mango008047) are involved in hemicellulose and pectin remodeling, suggesting active cell wall reinforcement critical for structural integrity. Conversely, downregulated genes such as *CSLB4* (mango018370), *PME20* (mango027211), *PMEI* (mango034748), and an additional *XTH23* isoform (mango011680) point to reduced hemicellulose synthesis and pectin demethylation, which would restrict excessive wall loosening. This coordinated balance between reinforcement and limited disassembly likely underlies the observed increase in firmness, allowing ripening delay without compromising texture.

### 3.6 Characterization of significantly altered transcription factors

**Fig. 8:**
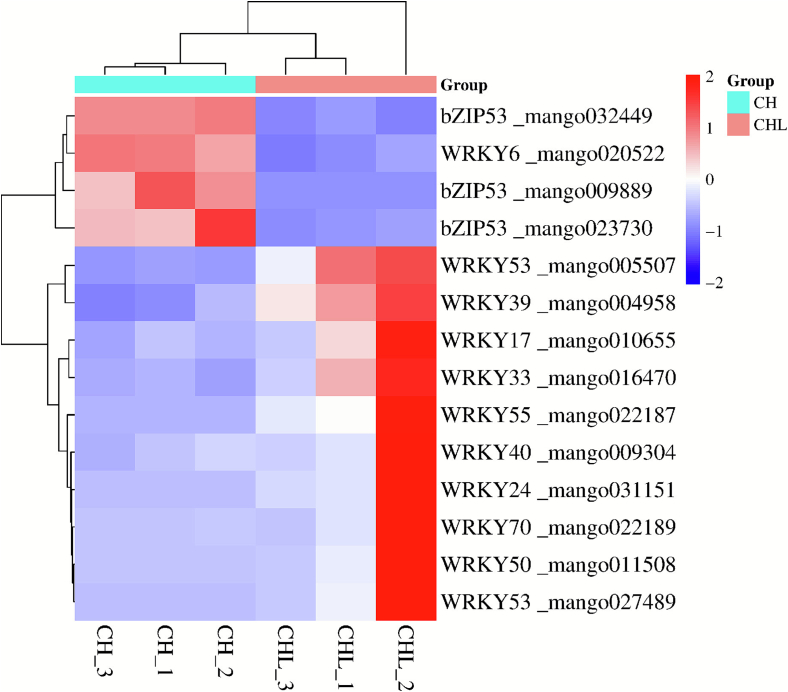
Differential expression of transcription factor families between CHL- and CH-treated mangoes.

A total of 66 DNA-binding transcription factors were identified, of which 34 DEGs were regulated (*p* ≤ 0.01, |log_2_FC| ≥ 2), belonging primarily to the WRKY and bZIP transcription factor families. Among the WRKY family, the majority (10 out of 11) were upregulated, with only one gene, *WRKY6* (mango020522), being downregulated. Additionally, all three identified *bZIP* transcription factors were downregulated (Fig. 8).

Heatmap showing the expression patterns of 14 significantly differentially expressed transcription factors (TFs) (p ≤ 0.01, |log_2_FC| ≥ 2), primarily from the WRKY and basic leucine zipper (bZIP) families. The majority of WRKY TFs are upregulated, while all identified bZIP TFs are downregulated in CHL compared to CH. Data are z-score normalized log2(TPM + 1) values from three biological replicates.

### 3.7 Hormone-related signal transduction pathways

A total of 53 hormone signal transduction-related genes were identified, displaying significant changes in auxin, ABA, and ethylene pathways following LPE treatment (*p*-value ≤0.01, |log_2_FC| ≥ 1.5). These genes exhibited distinct expression profiles under natural growth conditions across preharvest (40, 60, 80 DAF) and postharvest (4, 8, 12 DAP) stages, providing deeper insights into the mechanism by which LPE delays ripening.

**Fig. 9:**
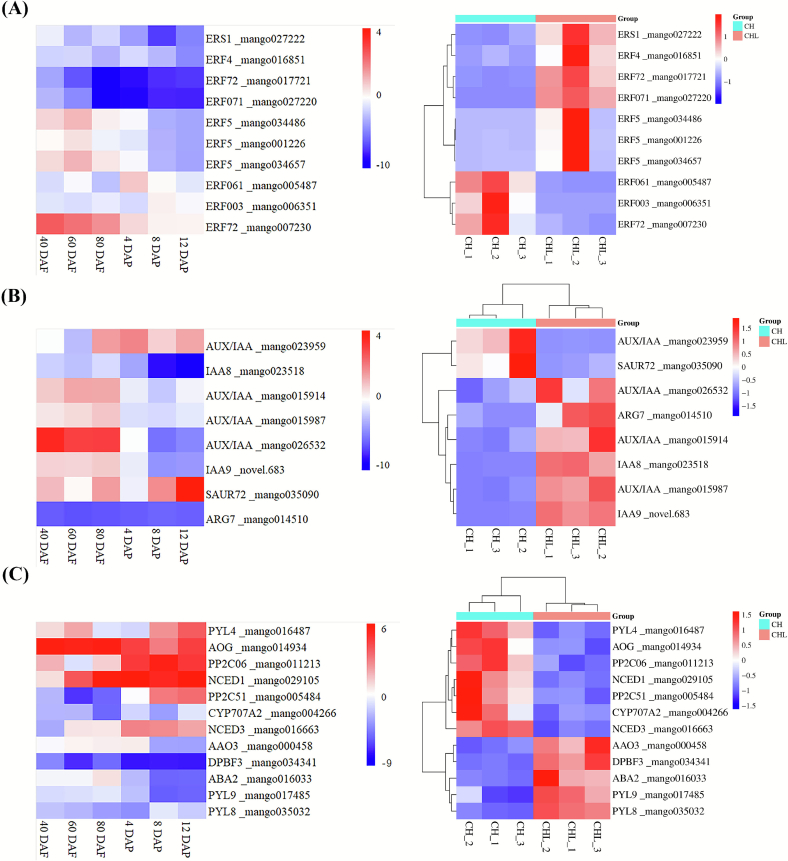
Expression patterns of hormone-related genes under natural conditions (left) and LPE treatment (right). (A) Ethylene-related genes: ethylene response sensor 1 (ERS1), ripening-associated ERFs (ERF003, ERF061), and stress-responsive ERFs (ERF4, ERF72, ERF5). (B) Auxin-related genes: auxin-responsive proteins (IAA8, IAA9) and a small auxin-up RNA (SAUR72). (C) Abscisic acid (ABA)-related genes: biosynthesis enzymes (NCED1, NCED3) and signaling receptors (PYL8, PYL9). Left panels show expression trends during natural development and postharvest ripening (40, 60, 80 days after flowering (DAF) and 4, 8, 12 days after postharvest (DAP)). Right panels show expression in response to CHL treatment on day 4 DAP. Data are normalized expression values. Red indicates upregulation, blue indicates downregulation relative to the respective control.

Under natural conditions (Fig. 9A, *right* panel), *ERS1* (mango027222) expression remains relatively stable from preharvest (40, 60, 80 DAF) and through postharvest (4, 8, 12 DAP) stages. This contrasted with ripening-related genes such as *ERF003* and *ERF061*, which were significantly upregulated postharvest. Under LPE treatment (right panel), *ERS1* expression was enhanced, suggesting maintained ethylene perception without triggering ripening. Concurrently, ripening-related transcription factors (*ERF003*, *ERF061*) were downregulated, while stress- and/or development-responsive ERFs (e.g., *ERF4*, *ERF72*, *ERF5*) were upregulated. This pattern indicates that LPE modulates ethylene sensitivity to favor developmental stability and stress adaptation over ripening processes.

Under natural conditions, auxin-responsive genes such as *IAA8* and *IAA9* were highly expressed during preharvest stages but declined postharvest, whereas the negative regulators *SAUR72* was upregulated, reflecting a reduction in auxin activity as ripening advanced. LPE treatment reversed this trend, notably upregulating *IAA8*, *IAA9*, and other auxin-responsive genes during postharvest stages while downregulating *SAUR72*. This change suggests that LPE sustains auxin activity, thereby promoting developmental processes and delaying the transition to ripening.

In natural conditions, ABA biosynthetic genes (*NCED1*, *NCED3*) were upregulated, and signaling regulators (*PYL8*, *PYL9*) were downregulated during postharvest stages. LPE treatment strongly downregulated *NCED1* and *NCED3*, thereby reducing ABA biosynthesis, while upregulating *PYL8* and *PYL9*, which maintains ABA sensitivity without excessive hormone accumulation. This indicates that LPE suppresses ABA-driven ripening while preserving stress-related ABA signaling capacity.

### 3.8 Validation of gene expression by qRT-PCR

**Fig. 10:**
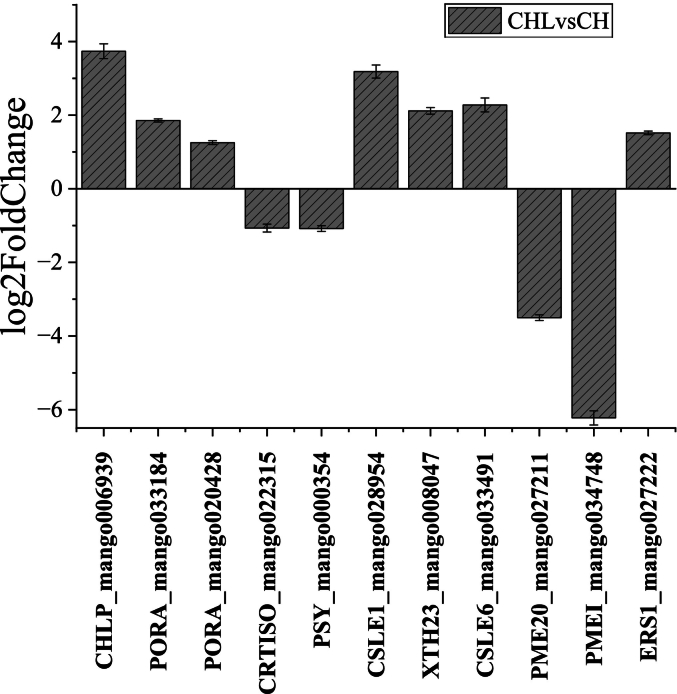
qRT-PCR validation of RNA-seq results for key ripening-related genes.

qRT-PCR analysis validated the RNA-seq expression patterns for 11 genes involved in cell wall remodeling, pigment dynamics, and ethylene signaling. Key hemicellulose crosslinking genes (*CSLE1*, *XTH23*, *CSLE6*) exhibited significant upregulation (log_2_FC = 3.18, 2.12, 2.28), consistent with the observed delay in fruit softening and chloroplast retention. Conversely, genes associated with pectin degradation (*PME20*, *PMEI*; log_2_FC = −3.50, −6.22) and carotenoid biosynthesis (*CRTISO*, *PSY*; log_2_FC = −1.07, −1.08) were suppressed, which aligns with the reduction in peel yellowing. The enhanced expression of the ethylene receptor gene *ERS1* (log_2_FC = 1.52) further indicated plasticity in ethylene signaling. These results provide robustly experimental validation for the the multi-targeted regulatory role of LPE in delaying mango ripening, as revealed by transcriptomic analysis (Fig. 10).

The log2 fold change (CHL/CH) values for 11 selected genes related to cell wall remodeling (CSLE1, XTH23, CSLE6, PME20, PMEI), pigment dynamics (CRTISO, PSY), and ethylene signaling (ERS1) as determined by quantitative real-time PCR (qRT-PCR). Data confirm the transcriptomic trends. Error bars represent standard error (SE) of the mean from three independent biological replicates, each with three technical replicates. The actin gene was used as an internal reference.

### 3.9 Correlations between hormone-related gene expression and fruit quality attributes

To statistically evaluate the link between hormonal transcriptional changes and the physiological outcomes of CHL treatment, Spearman correlation analysis was performed across all samples (*n* = 6) using the expression levels of 12 key hormone-related DEGs and 6 quality attributes. The analysis revealed several strong and statistically significant associations (*p* < 0.05) that align with the proposed mechanistic model (Supplementary Fig. S2, Supplementary Table S3). The expression of ripening-promoting genes *ERF003* and *ERF061* showed strong positive correlations with the yellowing rate (*r* = 0.88, *p* = 0.021 and *r* = 0.89, *p* = 0.019, respectively) and negative correlations with firmness. Conversely, genes upregulated by LPE exhibited protective associations: *ERF72* and *IAA9* expression was positively correlated with firmness (r = 0.89, p = 0.019 and *r* = 0.94, *p* = 0.005) and negatively with yellowing rate. Notably, *IAA8* expression was strongly negatively correlated with yellowing rate (*r* = −0.94, p = 0.005). Furthermore, *ERF4* and *PYL8/PYL9* expression was positively correlated with the retention of chelator-soluble pectin (CSP) (r = 0.94, p = 0.005 and *r* = 0.77, *p* = 0.072, respectively), a marker of cell wall integrity.

These significant correlations provide quantitative support that the variation in expression of specific hormone pathway genes is directly associated with the variation in key ripening indicators, reinforcing their functional relevance in the preservation phenotype induced by LPE.

## 4 Discussion

### 4.1 Multi-targeted regulation of cell wall integrity and pigment dynamics

The pursuit of sustainable postharvest solutions has intensified the exploration of agricultural by-products. Lychee peel extract (LPE), rich in polyphenols, has been primarily studied for its role in green nanotechnology and pharmacological effects, such as synthesizing nanoparticles and ameliorating metabolic syndromes in animal models. However, its direct application as a bioactive component in fruit preservation coatings, and the underlying molecular mechanisms, remain largely unexplored. Our study addresses this gap by revealing that LPE, in synergy with chitosan, orchestrates a multi-targeted intervention to delay mango ripening.

Specifically, the synergistic application of CHL delayed mango fruit softening through coordinated modulation of cell wall remodeling enzymes and pigment metabolism, establishing a dual mechanism for postharvest quality preservation. CHL-treated fruits exhibited reduced WSP accumulation and slower declines in CSP and HSP, indicative of stabilized pectin networks. Transcriptional upregulation of *CSLE1*, *CSLE6*, and *XTH23*, critical for hemicellulose crosslinking and pectin remodeling, aligns with conserved mechanisms in tomato and persimmon, where silencing these genes delays softening (Han et al., 2015). Conversely, suppressed *PME20* and *PMEI* expression modulated pectin methylesterase (PME) activity, preserving calcium-mediated crosslinking and cell wall rigidity (Wen et al., 2020; Xue et al., 2020). This dual regulation of cell wall dynamics not only delayed textural degradation but also enhanced pathogen resistance, as evidenced by the upregulation of *chitinase* genes (mango001735, mango007736) and *Mlo* genes (mango001574, mango002743) associated with defense responses (Salzman et al., 1998).

Concurrently, CHL treatment orchestrated chlorophyll retention and carotenoid suppression to decouple peel senescence from ripening progression. Upregulation of chlorophyll biosynthesis genes (*CHLP*, *PORA*) and downregulation of degradation regulators (*SGR*, *NYC1*) mirrored findings in tomato *sgr* mutants, where chlorophyll retention delays degreening (Cui et al., 2024). Similarly, suppressed expression of carotenoid biosynthetic genes (*CRTISO*, *PSY*) aligned with reduced lycopene accumulation in *CRTISO*-silenced tomatoes (Fantini et al., 2013; Gady et al., 2012). This transcriptional reprogramming highlights LPE's capacity to stabilize chloroplast functionality while suppressing chromatic transitions, thereby preserving visual freshness and extending marketability.

### 4.2 Metabolic reprogramming via organic acid enrichment and hormonal crosstalk

Integrated Metabolomic and transcriptomic analysis revealed that LPE-driven enrichment of organic acid, notably GABA, succinic acid, and glutaric acid, helps sustain pH homeostasis and attenuate ethylene biosynthesis. An enhanced glycolytic-TCA flux, indicated by the upregulation of *PDC2*, *CAC3*, and *GAPC1*, redirected carbon resources toward organic acid production rather than sugar accumulation, thereby helping to sustain titratable acidity (TA) levels (García-Gómez et al., 2020; Huang et al., 2016). The dual role of GABA in suppressing ethylene synthesis (via *ACS* and *ACO*) and enhancing antioxidant activity (Wu et al., 2024) further underscores its central importance in delaying ripening. This metabolic shift, together with transcriptional repression of sucrose/starch biosynthesis genes (e.g., *SPS1*, *AGPase S*), establishes a framework in which energy metabolism and acid retention **act** synergistically to decelerate senescence.

Analysis of hormonal crosstalk elucidated the nuanced regulation of ethylene, auxin, and ABA pathways by LPE. Although the ethylene receptor *ERS1* was upregulated, canonical ripening-associated *ERF003/ERF061* were suppressed, suggesting a plasticity in ethylene signaling similar to that in non-climacteric mechanisms (Huang et al., 2024; Pech et al., 2008). Auxin homeostasis was recalibrated through sustained expression of *IAA8/IAA9* and downregulation of *SAUR72*, mirroring the role of exogenous auxin in preserving cell wall integrity in strawberry and peach (Castro et al., 2021; Li et al., 2025; Zhao et al., 2023). ABA dynamics were modulated by suppression of *NCED1/3* and upregulation of *PYL8/9*, which attenuating ABA-driven ripening while preserving stress-responsive signaling (Ji et al., 2012, Ji et al., 2014; Moya-León et al., 2023; Zhang et al., 2009). This tripartite hormonal regulation demonstrates the ability of LPE to decouple maturation-associated transcriptional cascades from adaptive physiological responses.

Importantly, our correlation analysis provides direct statistical support for the physiological impact of these transcriptional changes. The expression levels of LPE-induced genes (e.g., *ERF72*, *IAA9*, *PYL8*) were significantly positively correlated with firmness and CSP content, while negatively correlated with yellowing rate. Conversely, the expression of LPE-suppressed genes (e.g., *ERF003*, *ERF061*, *NCED1*) showed the opposite pattern, being positively correlated with yellowing (Supplementary Fig. S2). These robust associations underscore that the magnitude of change in these hormonal pathways is quantitatively linked to the degree of ripening delay, positioning hormonal reprogramming as a central mechanistic conduit for LPE's preservative effect.

### 4.3 WRKY and bZIP transcription factors as potential regulators of postharvest quality

Our transcriptomic data revealed that LPE treatment significantly altered the expression of key transcription factor (TF) families, notably upregulating most *WRKYs* and downregulating *bZIPs* (Fig. 8). While direct transcriptional regulation requires further validation (e.g., EMSA or dual-luciferase assays), the coordinated expression patterns of these TFs with downstream ripening-related genes suggest their potential central roles in the CHL-mediated delay of senescence.

To explore this possibility in silico, we performed promoter cis-element analysis on the DEGs related to cell wall metabolism (e.g., *PME20*, *XTH23*) and pigment metabolism (e.g., *SGR*, *PSY*). Notably, the promoters of these genes were enriched for W-box (TTGACC/T) and G-box (CACGTG) elements, which are known binding sites for WRKY and bZIP family TFs, respectively (Supplementary Table S4). This bioinformatic prediction provides preliminary support for a potential regulatory relationship between the observed TF expression changes and the transcription of key ripening genes.

Our inference is further bolstered by functional studies in other fruit systems. For instance, in banana, MaWRKY21/26 have been shown to directly bind to the promoters of cell wall-modifying genes and repress their expression, thereby delaying softening (Jia et al., 2022). Conversely, in apple, the bZIP TF MdZF-HD11 promotes pectin degradation by activating *Mdβ-GAL18* (Wang, Wu, et al., 2024). The upregulation of stress-responsive *WRKYs* and downregulation of specific bZIPs in our CHL-treated mangoes align with these established regulatory paradigms, suggesting a conserved, yet LPE-induced, transcriptional network that stabilizes cell walls and pigments.

Therefore, we propose that the differential expression of *WRKY* and *bZIP* TFs is a key component of LPE's molecular signature. Their expression changes are tightly associated with the inhibition of cell wall disassembly and pigment turnover, likely through influencing the transcription of genes harboring corresponding cis-elements in their promoters. Definitive proof of direct binding and regulatory hierarchy awaits future functional characterization of these mango TFs.

### 4.4 Analysis of the synergistic mechanism and comprehensive advantages

The significantly enhanced preservation effect of CHL compared to CH alone indicates a clear synergistic interaction. Based on our findings and existing literature, this synergy likely arises from the complementary actions of the two components at both the physicochemical and biological levels.

#### 4.4.1 Complementary actions at the physicochemical level

Chitosan primarily functions by forming a semi-permeable film that modifies the internal atmosphere of the fruit and provides antimicrobial activity (Ali et al., 2022; Wang, Yuan, et al., 2024). The addition of LPE may further optimize this physical barrier. Polyphenolic compounds, such as those abundant in LPE, have been reported to interact with chitosan molecules through hydrogen bonding and other forces, which can improve the mechanical properties and gas barrier performance of composite coatings (Liu et al., 2022; Zhou et al., 2021). Therefore, it is plausible that in the CHL coating, LPE contributes to forming a more effective barrier against moisture loss and gas exchange, thereby synergistically enhancing the baseline preservation effect provided by chitosan alone. This type of physical synergy has been observed in other chitosan-based composite coatings incorporating plant extracts (Qian et al., 2025).

#### 4.4.2 Synergistic regulation at the biochemical and molecular level

Beyond potential physical interactions, the key synergy identified in this study lies in the unique biological activity of LPE. While chitosan's action is largely external and antimicrobial, our integrated transcriptomic and metabolomic analyses demonstrate that LPE actively modulates internal ripening pathways within the mango fruit. Specifically, CHL treatment (compared to CH) led to: (1) upregulation of cell wall strengthening genes (*CSLE1*, *XTH23*) and suppression of pectin degradation genes (*PME20*, *PMEI*); (2) enhanced expression of chlorophyll biosynthesis genes (*CHLP*) and downregulation of chlorophyll degradation (*SGR*) and carotenoid synthesis genes (*CRTISO*, *PSY*); (3) reprogramming of organic acid metabolism leading to GABA and succinate accumulation; and (4) altered hormone signal transduction, including downregulation of ripening-promoting transcription factors (*ERF003*, *bZIPs*) and upregulation of stress-related *WRKYs*. These multi-targeted regulatory effects, which were minimal or absent in the CH treatment, indicate that LPE components can influence the host fruit's gene expression network to delay senescence. This mechanism aligns with the concept that certain plant extracts, when combined with chitosan, can induce host defense or delay physiological processes (Saurabh et al., 2019; Shi et al., 2018).

#### 4.4.3 Comprehensive advantages stemming from the synergistic mechanism

The dual-layer synergistic mechanism outlined above provides a foundation for the comprehensive advantages of the CHL coating. In terms of preservation efficacy, the combination of an enhanced physical barrier and direct molecular intervention in ripening pathways explains why CHL outperforms single-component treatments like chitosan alone (Wang, Wu, et al., 2024) or preservatives targeting only ethylene action (Hofman et al., 2001). Regarding safety and sustainability, the mechanism relies entirely on natural, biodegradable components. Unlike some composite coatings incorporating synthetic agents or metal nanoparticles (Mwakalesi & Umbayda, 2024; Yuan et al., 2023), CHL utilizes food-grade materials and transforms agricultural waste (lychee peel) into a value-added product, supporting eco-friendly practices (Abdalla et al., 2023; Sun et al., 2023). Finally, the mechanism underpins its economic viability. The use of a low-cost byproduct (LPE) to significantly boost the efficacy of chitosan makes the formula cost-effective for potential large-scale application, offering a practical advantage over coatings requiring expensive commercial extracts (Alav et al., 2024).

In summary, the synergy between chitosan and LPE can be understood as a combination where chitosan provides foundational physical and antimicrobial protection, while LPE adds a layer of bioactive regulation that targets the fruit's internal ripening physiology. This cooperative action results in a preservation strategy that is more effective, sustainable, and economically feasible than many existing alternatives.

## 5 Conclusion

The CHL formulation represents a multi-targeted preservation strategy that integrates mechanisms of cell wall stabilization, metabolic recalibration, and hormonal plasticity. Our findings demonstrate that LPE significantly enhances the efficacy of chitosan. Firstly, LPE upregulates cell wall remodeling genes (*CSLE1*, *XTH23*) to stabilize pectin architecture, retaining fruit firmness. Secondly, it promotes chlorophyll retention by suppressing via *CRTISO* and *PSY*, thereby delaying peel yellowing. Thirdly, LPE decouples sugar-acid dynamics via the accumulation of GABA and succinic acid, which helps sustain metabolic homeostasis. Additionally, LPE attenuates ethylene-auxin-ABA crosstalk through the downregulation of ripening-specific transcription factors (*ERF003*, *bZIPs*) and the activation of stress-responsive WRKYs. Collectively, these actions enable LPE to extend mango shelf life without compromising quality, representing a paradigm shift for tropical fruit supply chains. Future studies should focus on evaluating the scalability of LPE across different cultivars and storage conditions, and further explore its dual antioxidant-antimicrobial properties for broader postharvest applications.

## CRediT authorship contribution statement

**Zhiwei Wu:** Writing – original draft, Visualization, Methodology, Investigation, Conceptualization. **Qinghua Qiao:** Writing – original draft, Visualization, Methodology, Investigation, Data curation. **Zhen Wang:** Validation, Methodology, Investigation, Data curation. **Tiancui Shang:** Validation, Resources, Investigation. **Shifang Wu:** Resources, Investigation. **PengPeng He:** Resources, Investigation. **Zhisheng Lin:** Validation, Investigation. **Zhenxin Ren:** Writing – review & editing, Supervision, Project administration, Funding acquisition, Conceptualization.

## Declaration of competing interest

The authors declare that they have no known competing financial interests or personal relationships that could have appeared to influence the work reported in this paper.

## Data Availability

Data will be made available on request.

## Appendix A Supplementary data

Supplementary material 1 Image 1

Supplementary material 2 Image 2

Supplementary material 3 Image 3

Supplementary material 4 Image 4

Supplementary material 5 Image 5

Supplementary material 6 Image 6
