# Supplementary material for: Lychee peel extract and chitosan synergistically delay mango ripening: Molecular insights
Source: Food Chem (Oxf). 2026 Jan 13;12:100355. doi: 10.1016/j.fochms.2026.100355 (PMC12874597; doi:10.1016/j.fochms.2026.100355)
Supplement: Supplementary file 1 — Supplementary material 1 [file mmc1.docx]

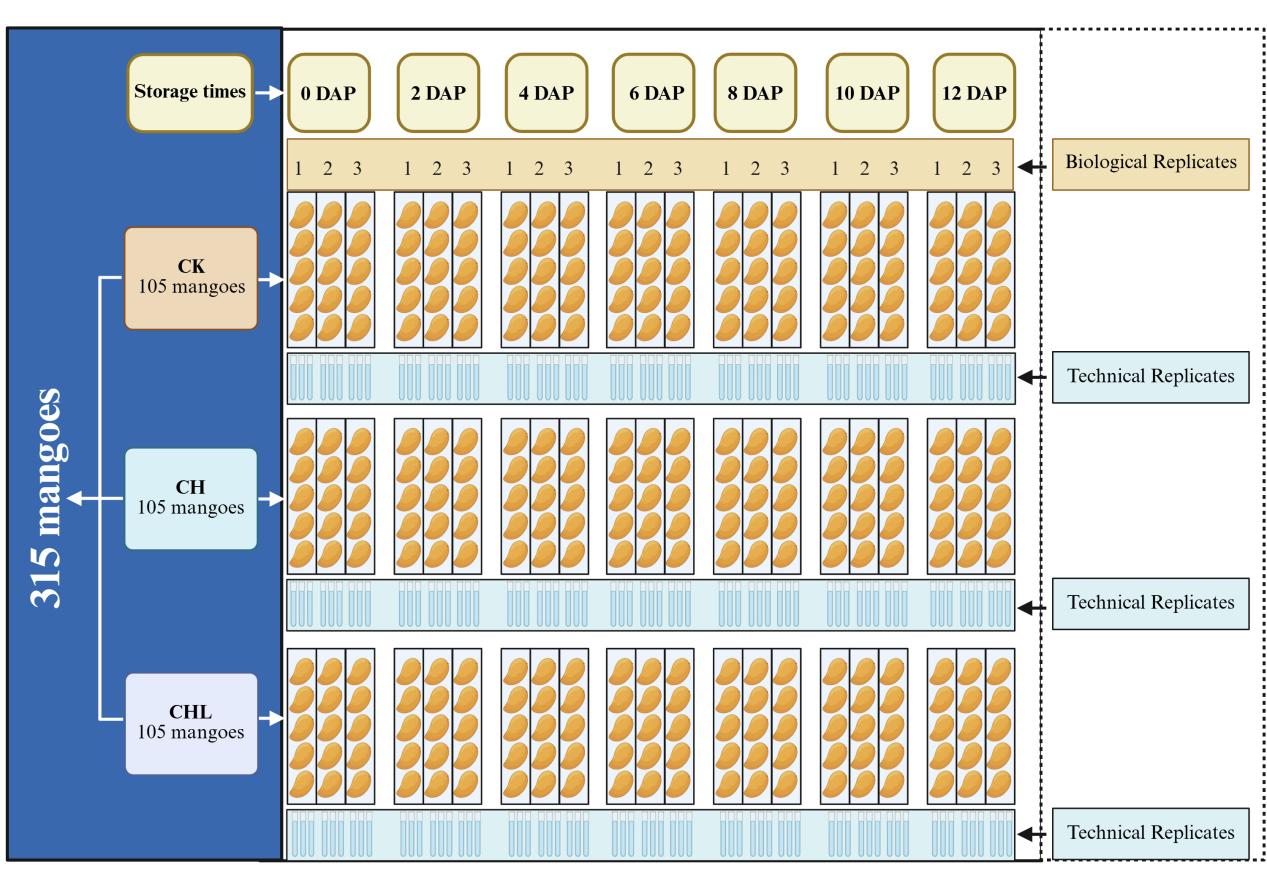


Fig. S1. Schematic overview of the experimental design, replication strategy, and sampling timeline.

Graphical representation of the experimental workflow used to evaluate postharvest treatments. A total of 315 mango fruits were initially allocated into three treatment groups: Control (CK), Chitosan (CH), and Chitosan-LPE (CHL), with 105 fruits per group. The study comprised seven postharvest storage time points (Days After Postharvest, DAP: 0, 2, 4, 6, 8, 10, 12). At each DAP, samples were drawn from each treatment group for analysis. The diagram illustrates the hierarchical replication structure: for a given treatment at a given DAP, sampling yielded three independent biological replicates (labeled 1, 2, 3). Subsequently, measurements on the samples from each biological replicate were performed with technical replicates (as indicated) to ensure analytical precision. This schema underlies all data collection for physiological, sensory, metabolomic, and transcriptomic analyses reported in the study.
