# Supplementary material for: Lychee peel extract and chitosan synergistically delay mango ripening: Molecular insights
Source: Food Chem (Oxf). 2026 Jan 13;12:100355. doi: 10.1016/j.fochms.2026.100355 (PMC12874597; doi:10.1016/j.fochms.2026.100355)
Supplement: Supplementary file 2 — Supplementary material 2 [file mmc2.docx]

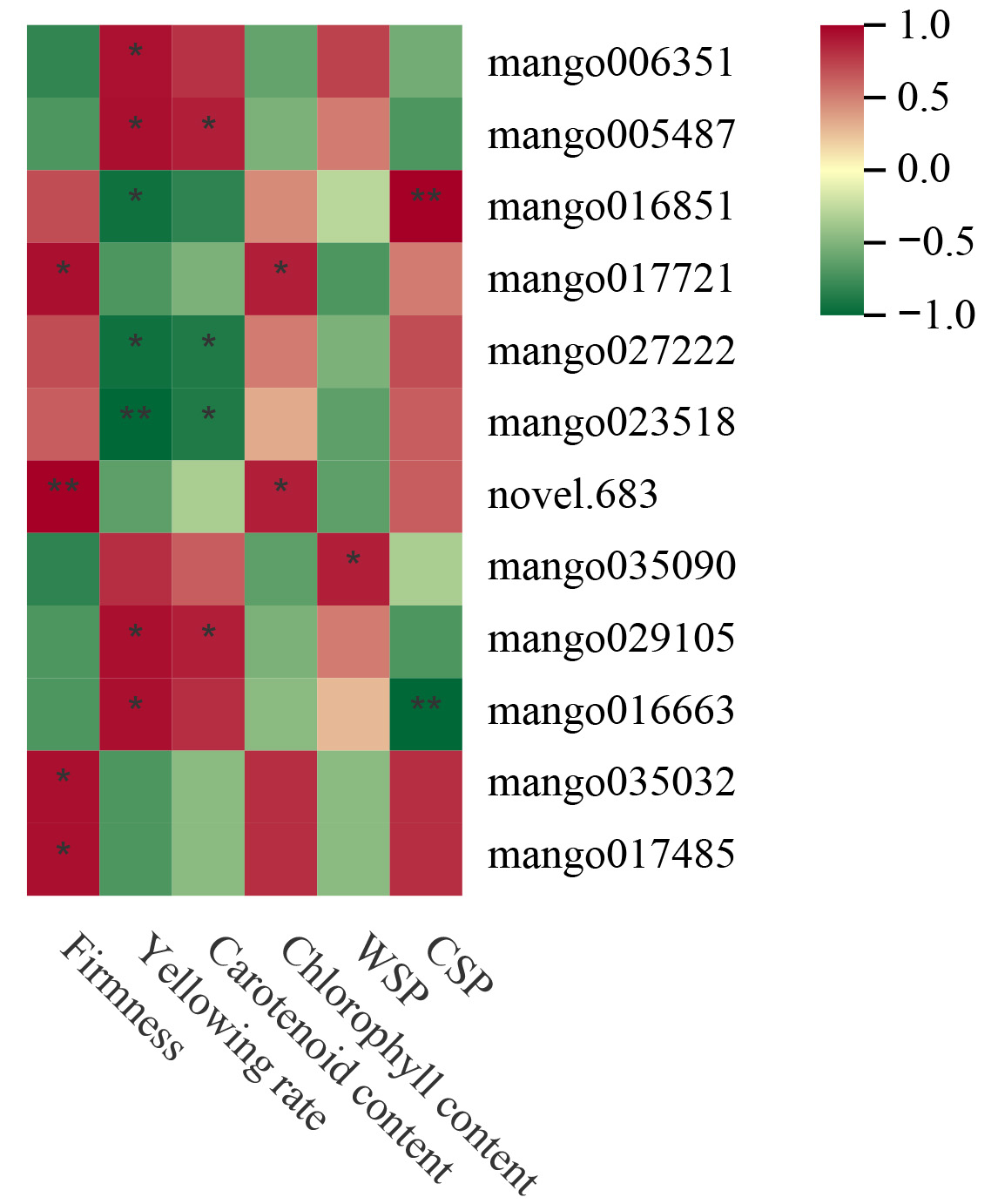


Figure S2. Correlation analysis between hormone-related gene expression and fruit quality attributes.

Spearman correlation heatmap illustrating the relationships between the expression levels of 12 key hormone-related differentially expressed genes (DEGs) (rows) and 6 critical fruit quality parameters (columns) across all samples (n=6). Correlation coefficients (r) are shown in cells, with color intensity indicating the strength and direction of correlation (red for positive, blue for negative). Asterisks denote statistical significance (*p < 0.05, **p < 0.01). Quality attributes: Firmness (N), Yellowing rate (%), Decay rate (%), Soluble solids content (SSC, %), Titratable acidity (TA, %), Chelator-soluble pectin content (CSP, mg/g).
